# Taste, Nutrition, and Health

**DOI:** 10.3390/nu12010155

**Published:** 2020-01-06

**Authors:** Beverly J Tepper, Iole Tomassini Barbarossa

**Affiliations:** 1 Department of Food Science, School of Environmental and Biological Sciences, Rutgers University, New Brunswick, NJ 08901-8520, USA; 2 Department of Biomedical Sciences, University of Cagliari, 09042 Monserrato, Italy; tomassin@unica.it

## Abstract

The sensation of flavour reflects the complex integration of aroma, taste, texture, and chemesthetic (oral and nasal irritation cues) from a food or food component. Flavour is a major determinant of food palatability—the extent to which a food is accepted or rejected—and can profoundly influence diet selection, nutrition, and health. Despite recent progress, there are still gaps in knowledge on how taste and flavour cues are detected at the periphery, conveyed by the brainstem to higher cortical levels and then interpreted as a conscious sensation. Taste signals are also projected to central feeding centers where they can regulate hunger and fullness. Individual differences in sensory perceptions are also well known and can arise from genetic variation, environmental causes, or a variety of metabolic diseases, such as obesity, metabolic syndrome, and cancer. Genetic taste/smell variation could predispose individuals to these same diseases. Recent findings have also opened new avenues of inquiry, suggesting that fatty acids and carbohydrates may provide nutrient-specific signals informing the gut and brain of the nature of the ingested nutrients. This special issue on “Taste, Nutrition, and Health” presents original research communications and comprehensive reviews on topics of broad interest to researchers and educators in sensory science, nutrition, physiology, public health, and health care.

## 1. Sweet Taste

Understanding the role of sweet taste in health and nutrition has been a major focus of chemosensory research for more than 50 years. Although significant strides have been made in this area, a complete understanding of the complex links between sweet taste perception, liking, and intake remains elusive. Tan and Tucker [1] reviewed the current state of knowledge in this area, concluding that current measures of sweet taste perception and liking may have limited capacity to predict dietary behaviours. The characterization of individuals as “sweet likers” or “sweet dislikers” has been a useful concept for understanding person-to-person differences in hedonic reactions to sweetness across a range of intensities. Building on their previous work, Iatridi, Hayes, and Yeomans [2] presented a new methodological approach for fine-tuning sweet-liker/-disliker classifications. These advances are taking place against a backdrop of escalating public health concerns about excess sugar in the diet and are reflected in current dietary guidelines in the United States [3] and many other countries across the globe [4], which now limit daily sugar consumption. To achieve the goal of sugar reduction at the population level, consumers would need to change their behaviours by making different diet choices, selecting sugar-reduced products, or a combination of these activities. Sugar reduction has been an ongoing focus of the food industry. Wee, Tan, and Forde’s [5] study of 16 sweeteners provides an up-to-date and comprehensive guide for comparing the potencies of several classes of sweeteners to sucrose, the goal standard. Sweetener classes include, e.g., saccharides and polyols, non-nutritive synthetics (e.g., aspartame, sucralose), and non-nutritive naturals such as stevia.

## 2. Food Preferences/Individual Differences

Understanding individual differences in food preferences and eating behaviours has important implications for both food research and nutrition monitoring. Many of the contributions in this issue examine individual differences, from a variety of perspectives such as age, gender, culture/ethnicity, and genetic variation. For example, to gain insight into food preferences in a cross-cultural context, Wanich et al. [6] compared liking ratings for foods tasted in the laboratory to general liking responses obtained by questionnaire. Jilani et al. [7] studied a large European family cohort (>12,000 respondents) to establish the validity of a single instrument collecting food preference data from children, adolescents, and adults. The review by Keller et al. [8] presents a new conceptual model and fresh look at sex differences in eating behaviours in children. Two papers address the role of genetic variation in food preferences and choice. De Toffoli et al. [9] examined the interaction between PROP taste sensitivity (a marker for bitter taste) and psychological traits on the selection of astringent, polyphenol-rich foods, while the short review by Robino et al. [10] proposes that other genes and phenotypes (in addition to traditional taste-modifying genes) may play a role in food preferences.

## 3. Umami and Fat Taste

The role of other taste sensations in nutrition and health remains a vibrant and active area of research interest. Two contributions in this issue focus on fatty acid taste sensations. Sollai et al. [11] utilized a novel technique to measure electrophysiological responses from the gustatory cells of the human tongue following the direct application of oleic acid. They report strong associations between physiological signals and self-reports of fat taste sensations, demonstrating the reliability of this technique. Furthermore, Peterschmitt et al. [12] showed that direct lingual application of long-chain fatty acid to the circumvallate papillae of the mouse activated brain circuits involved in taste signaling, reward, and memory. Together, these studies reveal important features of the gustatory, peripheral, and central mechanisms involved in fat taste that are relevant to both animals and humans.

Finally, Hartley, Liem, and Keast [13] re-examine the notion that umami qualifies as a basic taste. They argue that umami meets most of the criteria for a basic taste—it is elicited by a distinct class of stimuli (e.g., L-glutamate), it activates specific receptor(s), (e.g., T1R1/T1R3), etc., but it does not generate a unique taste quality. They propose a new subclassification called “alimentary taste” for umami, and other taste qualities (such as fat) that may be more important signals for regulating postingestive metabolism than as sensory cues for the presence of specific nutrients in foods.

## 4. Disease States and Role of the Gut

Alterations in taste or smell are well-known features of a variety of metabolic diseases and pathological states. However, for many of these conditions, data from well-described clinical populations are scarce. In this issue, Singh et al. [14] present comprehensive findings on taste disruptions and oral complaints in patients with Sjögren’s syndrome, an autoimmune disease affecting exocrine glands, such as the salivary glands, which results in dry mouth, burning mouth, and poor oral health. Importantly, this study included patients with Sjögren’s syndrome, individuals with so-called “sicca” complaints who do not meet the diagnostic criteria for the disease (and are rarely studied), and healthy controls. There is also a critical need to develop food products that help patients with nutritional diseases to adhere to prescribed diets. Proserpio et al. [15] assessed the acceptability of different formulations of low-phenylalanine foods using a check-all-that-apply (CATA) methodology in individuals with phenylketonuria.

Obesity is increasingly characterized as an inflammatory disease arising from gut dysbiosis associated with an obesogenic diet. In the study by Bernard et al. [16], mice chronically fed a high-fat diet exhibited a blunted preference for sucrose that was partially corrected by supplementing the diet with a prebiotic (10% inulin-type fructan). Examination of caecal contents showed a greater abundance of beneficial bacteria in the diet-induced obese mice fed the prebiotic supplement. These interesting findings suggest that prebiotic supplementation warrants more attention as an aid to the dietary management of obesity.

Lastly, taste receptors are expressed throughout the gastrointestinal tract and are known to release satiety hormones such as GLP-1, CCK, and PYY. In a single-blind, crossover trial, Klaassen et al. [17] delivered a tastant mixture via a naso-duodenal-ileal catheter to healthy participants and measured food intake and satiety from a subsequent meal. However, no differences in outcome measures were observed as a function of duodenal (proximal) or ileal (distal) infusions.

## 5. Lifestyle Factors

Two papers examine the extent to which lifestyle factors influence taste perception and food preferences in healthy individuals. Using fMRI, Gramling, Kapoulea, and Murphy [18] demonstrate that chronic caffeine consumers and nonconsumers experience differential activation in neuronal areas involved in reward, memory, and information processing when they are exposed to bitter and sweet tastants. Likewise, Feeney et al. [19] showed that in men, habitual physical activity selectively alters taste perceptions. Specifically, active men gave higher intensity ratings to sweet and umami solutions in comparison to nonactive men.

The study by Larsen et al. [20] examined the complex interrelationships between taste and diet in a cohort of chronic smokers who were also overweight or obese. Because obese smokers reportedly use smoking as a means of controlling their appetite and weight [21], gaining greater insights into taste changes and smoking-related dietary behaviors in this population may have important implications for treatment and prevention. Notably, participants also rated a liking for sweet e-juice, which is used to flavor e-cigarettes, a popular alternative to tobacco cigarettes. Using structural modeling, Larsen et al. [20] showed that taste (including e-juice liking) was associated with body mass index (BMI) in chronic smokers through liking of fats/carbohydrates and that smoking-related dietary behaviors (assessed by questionnaire) could influence BMI by a separate pathway. These novel findings could help to inform the development of new smoking intervention strategies.

## 6. New Product Formulations

This volume would not be complete without addressing consumer acceptance of new products and formulations designed to enhance health and wellbeing. Grapefruit is rich in vitamins, antioxidants, and anti-inflammatory compounds, but is rejected by many consumers due to its bitter taste. Gous et al. [22] developed 36 model grapefruit beverages varying in taste, aroma, flavor, and color to characterize their sensory profiles and to identify the formulations best-liked by consumers. Franks et al. [23] present unique findings showing that the type of water (tap, bottled, or deionized) used to brew tea influences sensory characteristics and nutrient extraction. Color, flavor, and epigallocatechin gallate (EGCG) extraction were higher for teas (especially green tea) made with purified water, but consumer liking was higher for less intensely flavored green tea made with tap water. These findings suggest that the consumer’s choice of water source can maximize the flavor or health benefits of tea according to their personal preferences.

## 7. Olfaction

The determination of the odor detection threshold is a classic technique for assessing smell function, but such methodology is time-consuming and not well suited to diagnostic evaluation in the clinical setting or in the field with a large number of subjects. Using Sniffin’ Sticks (odour-impregnated pens) and a Bayesian adaptive algorithm (QUEST protocol), Höchenberger and Ohla [24] established a rapid method with reduced testing duration and less variability between measurements.

## References

[1] Tan S.-Y., Tucker R.M. (2019). Sweet Taste as a Predictor of Dietary Intake: A Systematic Review. Nutrients.

[2] Iatridi V., Hayes J.E., Yeomans M.R. (2019). Quantifying Sweet Taste Liker Phenotypes: Time for Some Consistency in the Classification Criteria. Nutrients.

[3] McGuire S. (2011). U.S. Department of Agriculture and U.S. Department of Health and Human Services, Dietary Guidelines for Americans, 2010. 7th Edition, Washington, DC: U.S. Government Printing Office, January 2011. Adv. Nutr..

[4] World Health Organization (2015). Guideline: Sugars Intake for Adults and Children.

[5] Wee M., Tan V., Forde C. (2018). A Comparison of Psychophysical Dose-Response Behaviour across 16 Sweeteners. Nutrients.

[6] Wanich U., Sayompark D., Riddell L., Cicerale S., Liem D.G., Mohebbi M., Macfarlane S., Keast R. (2018). Assessing Food Liking: Comparison of Food Liking Questionnaires and Direct Food Tasting in Two Cultures. Nutrients.

[7] Jilani H., Pohlabeln H., De Henauw S., Eiben G., Hunsberger M., Molnar D., Moreno L.A., Pala V., Russo P., Solea A. (2019). Relative Validity of a Food and Beverage Preference Questionnaire to Characterize Taste Phenotypes in Children Adolescents and Adults. Nutrients.

[8] Keller K.L., Kling S.M.R., Fuchs B., Pearce A.L., Reigh N.A., Masterson T., Hickok K. (2019). A Biopsychosocial Model of Sex Differences in Children’s Eating Behaviors. Nutrients.

[9] De Toffoli A., Spinelli S., Monteleone E., Arena E., Di Monaco R., Endrizzi I., Gallina Toschi T., Laureati M., Napolitano F., Torri L. (2019). Influences of Psychological Traits and PROP Taster Status on Familiarity with and Choice of Phenol-Rich Foods and Beverages. Nutrients.

[10] Robino A., Concas M.P., Catamo E., Gasparini P. (2019). A Brief Review of Genetic Approaches to the Study of Food Preferences: Current Knowledge and Future Directions. Nutrients.

[11] Sollai G., Melis M., Mastinu M., Pani D., Cosseddu P., Bonfiglio A., Crnjar R., Tepper B.J., Tomassini Barbarossa I. (2019). Human Tongue Electrophysiological Response to Oleic Acid and Its Associations with PROP Taster Status and the CD36 Polymorphism (rs1761667). Nutrients.

[12] Peterschmitt Y., Abdoul-Azize S., Murtaza B., Barbier M., Khan A.S., Millot J.-L., Khan N.A. (2018). Fatty Acid Lingual Application Activates Gustatory and Reward Brain Circuits in the Mouse. Nutrients.

[13] Hartley I.E., Liem D.G., Keast R. (2019). Umami as an ‘Alimentary’ Taste. A New Perspective on Taste Classification. Nutrients.

[14] Singh P.B., Young A., Homayouni A., Hove L.H., Petrovski B.É., Herlofson B.B., Palm Ø., Rykke M., Jensen J.L. (2019). Distorted Taste and Impaired Oral Health in Patients with Sicca Complaints. Nutrients.

[15] Proserpio C., Pagliarini E., Zuvadelli J., Paci S., Re Dionigi A., Banderali G., Cattaneo C., Verduci E. (2018). Exploring Drivers of Liking of Low-Phenylalanine Products in Subjects with Phenyilketonuria Using Check-All-That-Apply Method. Nutrients.

[16] Bernard A., Ancel D., Neyrinck A.M., Dastugue A., Bindels L.B., Delzenne N.M., Besnard P. (2019). A Preventive Prebiotic Supplementation Improves the Sweet Taste Perception in Diet-Induced Obese Mice. Nutrients.

[17] Klaassen T., Alleleyn A.M.E., van Avesaat M., Troost F.J., Keszthelyi D., Masclee A.A.M. (2019). Intraintestinal Delivery of Tastants Using a Naso-Duodenal-Ileal Catheter Does Not Influence Food Intake or Satiety. Nutrients.

[18] Gramling L., Kapoulea E., Murphy C. (2018). Taste Perception and Caffeine Consumption: An fMRI Study. Nutrients.

[19] Feeney E.L., Leacy L., O’Kelly M., Leacy N., Phelan A., Crowley L., Stynes E., de Casanove A., Horner K. (2019). Sweet and Umami Taste Perception Differs with Habitual Exercise in Males. Nutrients.

[20] Larsen B.A., Litt M.D., Huedo-Medina T.B., Duffy V.B. (2019). Modeling Associations between Chemosensation, Liking for Fats and Sweets, Dietary Behaviors and Body Mass Index in Chronic Smokers. Nutrients.

[21] Rupprecht L.E., Donny E.C., Sved A.F. (2015). Obese Smokers as a Potential Subpopulation of Risk in Tobacco Reduction Policy. Yale J. Biol. Med..

[22] Gous A.G.S., Almli V.L., Coetzee V., de Kock H.L. (2019). Effects of Varying the Color, Aroma, Bitter, and Sweet Levels of a Grapefruit-Like Model Beverage on the Sensory Properties and Liking of the Consumer. Nutrients.

[23] Franks M., Lawrence P., Abbaspourrad A., Dando R. (2019). The Influence of Water Composition on Flavor and Nutrient Extraction in Green and Black Tea. Nutrients.

[24] Höchenberger R., Ohla K. (2019). Estimation of Olfactory Sensitivity Using a Bayesian Adaptive Method. Nutrients.

